# Pectin Based Hydrogels for Drug Delivery Applications: A Mini Review

**DOI:** 10.3390/gels8120834

**Published:** 2022-12-17

**Authors:** Sung Soo Han, Seong Min Ji, Min Jung Park, Maduru Suneetha, Uluvangada Thammaiah Uthappa

**Affiliations:** 1 School of Chemical Engineering, Yeungnam University, 280 Daehak-Ro, Gyeongsan 38541, Republic of Korea; 2 Institute of Cell Culture, Yeungnam University, 280 Daehak-ro, Gyeongsan 38541, Republic of Korea

**Keywords:** hydrogels, pectins, controlled release, biopolymers, targeted drug delivery

## Abstract

Over the past few decades, hydrogel systems using natural polymers have been expansively employed in drug delivery applications. Among the various reported biopolymer-based hydrogel drug delivery systems, pectin (Pec) is an exceptional natural polymer due to its unique functionalities and excellent properties such as biocompatibility, biodegradability, low-cost, and simple gelling capability, which has received considerable interest in the drug delivery fields. Since there is an increasing need for biomaterials with unique properties for drug delivery applications, in this review, hydrogels fabricated from natural pectin polymers were thoroughly investigated. Additionally, the present mini review aims to bring collectively more concise ways such as sources, extraction, properties, and various forms of Pec based hydrogel drug delivery systems and their toxicity concerns are summarized. Finally, the potential objectives and challenges based on pectin-based hydrogel drug delivery systems are also discussed.

## 1. Introduction

Biomaterials with exceptional properties have gained a lot of study interest, specifically in drug delivery applications. Polymers, both synthetic and natural, are regarded as better candidates in the fabrication of biomaterials [1,2,3,4]. Hydrogels, films, nanoparticles, and nanocomposites are just a few of the drug formulations that have been designed and advanced in drug delivery fields [5,6,7,8]. Among the formulations above-mentioned, hydrogels have grown in popularity due to their intriguing properties such as biocompatibility, biodegradability, and exclusive “soft-wet” nature in correlation to biological tissue [9,10]. It is worthwhile mentioning that hydrogels have a high-water content, which could swell and adsorb liquid due to their porous nature, and an injectable hydrogel is highly efficient for clinical use. In terms of tumor application, hydrogels possess excellent biocompatibility and controllability, and some of these hydrogel systems are used in various other applications such as additives, the chemical industry, energy, and water treatment [11,12,13,14,15,16]. Despite these exceptional benefits, hydrogels in bio-related applications face some challenges due to limitations such as mechanical stiffness, water sensitivity, and instability in physiological conditions [17].

Polymeric materials are classified into two types: synthetic and natural. Because of their biodegradability and biocompatibility, natural polymers have distinct merits over synthetic polymer systems. The main disadvantages of using natural polymers are their low mechanical properties over synthetic polymers, which makes them unsuitable for a variety of biomedical applications. Several reports have specified that the most synthetic polymers have drawbacks such as high cytotoxicity and low biocompatibility [18,19]. Generally, biopolymers are comprised of monomeric units covalently attached to form bigger biomolecules. Usually, pectic substances are differentiated into four different types: protopectin, pectic acid, pectinic acid, and Pec [20]. Among the various biopolymers, Pec is a kind of water-soluble anionic heteropolysaccharide found from the primary cell walls of terrestrial plants extracted using chemical or enzymatic process [21]. Pec possesses a higher range of heterogeneity in their structure due to their sources and methods of different extraction process [22]. Pec is considered as a promising candidate in the drug delivery field due to its excellent features such as non-toxicity, biocompatibility, biodegradability, low-cost, antibacterial, and anti-inflammatory properties [23]. 

To our best of knowledge, recently there have been no reviews published specifically focusing on “Pec based hydrogel for drug delivery systems”. However, there are two general reviews on Pec based biomaterials for biomedical applications [23,24]. Thus, this is a sole mini review where we tried to compile the latest progress and advances specifically on Pec based hydrogels for drug delivery applications. Thus, this present mini review could target a wide audience/researchers who exclusively work on Pec and Pec based hydrogel systems. This mini review details the fabrication of hydrogels from natural Pec polymers and aims to collectively bring more concise ways such as sources, extraction, properties, and various forms of Pec based hydrogels in drug delivery applications and their toxicity concerns. Finally, the possible purposes and challenges based on Pec based hydrogel drug delivery systems are also discussed.

## 2. Sources

Some of the sources for Pec are from apple pomace, citrus peels, and, more recently, sugar beet pulp [25]. Certainly, tropical and subtropical fruit by-products are primarily a significant source of Pec. Nevertheless, it is important to mention that the Pec yield and physicochemical properties of Pec are affected by the extraction technique as well as additional variables such as the extraction time, type of acid, pH, temperature, and the liquid–solid ratios [26].

## 3. Pectin Extraction

The yield of extracted Pec as well as the quality can be used to evaluate the suitability of the extraction method because mass transfer into the extraction solvents governs Pec extraction. To extract Pec from natural sources, several methods have been used including traditional hot extraction and advanced procedures such as ultrasound, microwave, and enzymatic processes. Huge efforts are being made to promote “green” chemistry and technology. In terms of Pec extraction, hot conventional extraction necessitates a lengthy protocol, more energy, and the use of strong acids, which is contrary to “green” chemistry principles. Thus, an outline is depicted in the below section on conventional and non-conventional extraction methods.

### 3.1. Conventional Extraction

Extraction temperature, solid–liquid ratio, pH, particle size, and extraction time are all factors that influence the yield and quality of the extracted Pec. The utilization of mineral acids for Pec extraction is linked to environmental concerns as well as higher costs. Concerning the emergent concept of “green” chemistry and the drawbacks associated with the practice of mineral acids, the emphasis is now shifting to “food” compatible acids [27].

### 3.2. Ultrasound Mediated Extraction

Ultrasonic waves with frequencies from 20 to 100 kHz are commonly used. It is important to note that ultrasound frequency influences the extraction process because it influences the size of the microbubbles and their resistance to mass transfer. Furthermore, an upsurge in ultrasound frequency results is a decrease in the production and intensity of cavitation in liquid [28,29]. Several studies backing up the substantial assistance of the ultrasound-assisted extraction has several merits including low energy, less extraction time, minimal solvent, and enhanced extraction yield in support of using ultrasound as a “green” extraction method.

### 3.3. Microwave Mediated Extraction

This technique needs less processing time and solvent, and produces a higher extraction yield and generate superior qualities [30]. Microwave extraction is the process of applying a microwave field to a dielectric material. Ionic conduction and dipole rotation heat the solvent–sample matrix. Microwave energy initiates the electrophoretic transfer of ions and electrons, resulting in an electric field that drives particle movement, whereas dipole rotation is instigated by the substitute movement of polar molecules. Microwave power, measured in Watts (W), is a key factor in Pec extraction. Increased microwave power was found to be positively related to extraction efficiency [31].

### 3.4. Enzyme Aided Extraction

For enzyme-aided Pec extractions, enzymes must be able to display reactions with precise specificity and selectivity. Enzymes used in Pec extraction disturb features of the plant cell wall, enabling pectin release and reducing the complete extraction period [32]. There are more additional benefits of using enzyme aided extraction such as avoiding the corrosion of equipment by acids, reduced energy consumption, and the specificity of enzymes yield an improved quality of Pec [33]. 

### 3.5. Combination of Non-Conventional Technologies

Researchers have looked at how non-traditional extraction techniques combine to effectively extract Pec from tropical and subtropical fruit waste. Ultrasound-microwave-assisted extraction, which combines ultrasonic and microwave extraction approaches, is viewed as an efficient process [34,35]. Ultrasound-microwave-mediated extraction involves rapid yield and competent Pec extraction at low temperatures at ambient conditions, saving energy, time, and is economically viable [36].

## 4. Structure of Pec

**Figure 1:**
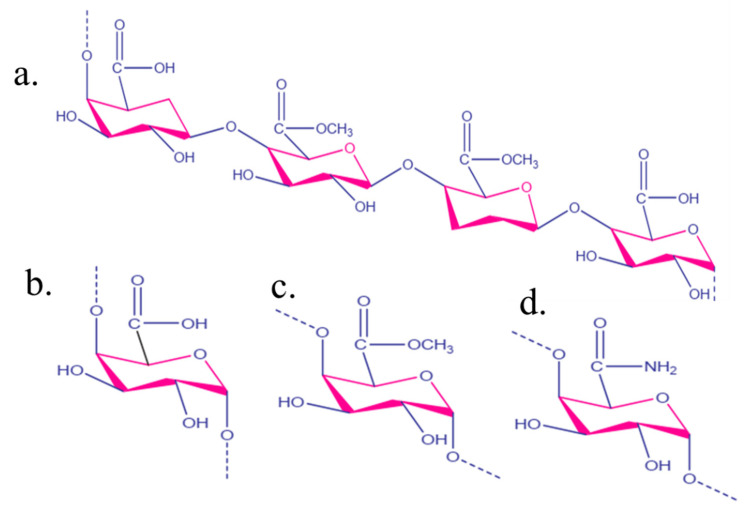
(**a**) The frequent unit of chemical moieties in the Pec chemical structure, (**b**) carboxylic, (**c**) ester, and (**d**) amide groups. Reproduced with permission [46].

**Figure 2:**
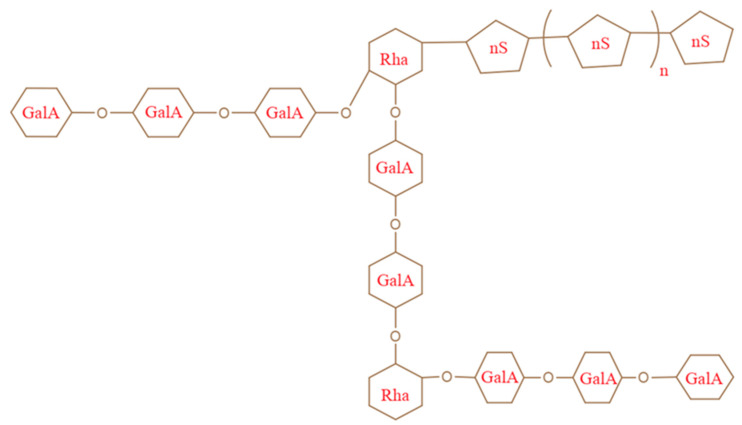
Rhamnose (Rha) insertion occurrence of galacturonic acid (Ga) and nS (neutral sugar). Reproduced with permission [46].

Pec is widely present in the cell walls of terrestrial/earthy plants [37]. Pec was made and explored in the powder form, which is very simple to use and handle [38]. Pec is recognized as a significant component of the middle lamella, which helps to keep cells organized. Every part of the plant contains different amounts of Pec and chemical assemblies. In terms of the chemical composition and molecular density, Pec in fruits and vegetables exists in poly-molecular and poly-disperse forms [39]. The monomeric units of Pec may vary depending on the sources, procedure used for separation, and successive examinations. Depending on the origin and method of isolation, diverse properties of Pec can be used to prepare its innumerable forms [40]. The Pec is comprised of chemical moieties such as the carboxylic (-COOH) group, ester and amide (-NH_2_) groups [41], as shown in (Figure 1). Figure 2 displays the representative overview of rhamnose addition, which leads to the existence of the galacturonic acid (GA) chain, where S indicates the presence of neutral sugars [42]. 

## 5. Physical-Chemical Properties of Pec

Pec is a class of substances that when it is dissolved in water under certain environments, it can form gels. It is obtained from protopectin, which is found in the plant cell middle lamellae [43]. All of Pec’s physical properties are due to its bi-linear poly-anion configuration (poly-carboxylate) [44]. When it comes to chemical features, the depolymerization of dissolved Pec occurs in aquatic classifications, and Pec has the highest stability at pH 4. The Pec de-esterifies below and above this pH, resulting in decreased stability. Depolymerization occurs at low pH levels via the acid catalyst hydrolysis of glycosidic bonds [45].

## 6. Application of Pec-Based Hydrogels in Drug Delivery

Over the last 50 years, hydrogels based on biodegradable natural polymers have been widely used in drug delivery systems [47]. Hydrogels have expanded in the drug delivery field as their three-dimensional structures have exclusive properties such as being hydrophilic in nature, biocompatibility, biodegradability, moist environments in surrounding tissues, and low cost [23,48]. Thus, in this section, various forms of Pec-based hydrogels in drug delivery applications are showcased.

In 2018, a low-density lipoprotein (LDL)-Pec nanogels in the presence of alginate hydrogel beads was successfully fabricated via the ionotropic gelation process. The designed LDL/pectin nanogels were confined in the core of the alginate-based hydrogel beads without affecting the properties of the hydrogel beads. Furthermore, curcumin (CUR) was encapsulated into the LDL-Pec nanogels to assess their role as pH dependent studies. The release of CUR was greatly prolonged by adding nanogels to alginate hydrogel beads, where the release profile of curcumin proved to have a slightly slower rate in the simulated GI conditions, signifying a role as an oral drug delivery system [49].

In another work, hydrogels were fabricated by graft polymerization and magnetic nanoparticles (MNPs) comprised in Pec-based hydrogels through the in situ method for the controlled release of the diclofenac sodium (DS) drug. Due to the presence of MNPs, there was a substantial enhancement in the mechanical properties, swelling capacity, drug loading efficiency, and drug release performances. Due to the porous network and high surface of the MNPs, the MNP-based pectin hydrogels displayed 68.84% of drug loading efficiency, however, without MNPs, the Pec-based hydrogels showed only 44.84% of loading efficiency. It was identified that around 95% of the DS drug was released from the MNP based Pec hydrogels, suggesting that swelling controlled the diffusion mode of the drug release profiles [50].

Other research investigations have been reported to improve the therapeutic effects and lessen the toxic effects of the systematic drug administration of vancomycin hydrochloride (vanco HCl), a hydrogel scaffold of silk fibroin/oxidized pectin (SF/OP) designed based on the Schiff-base reaction. To obtain a sustained release profile of the drug, electrospun fibers of poly(L-lactide) (PLLA) were incorporated into the hydrogel and unveiled high 97% of drug loading efficiency, followed by a 61% decrease in drug release content. For the first 24 h, the drug release profile from hydrogel was 39.97% and in the hydrogel/fiber system, a 13.83% decrease in the drug release content was observed, followed by promising sustained release up to 192 h. In addition, the designed hydrogel drug delivery systems proved to be non-toxic against human adipose derived mesenchymal stem cells (hAD-MSCs) [51].

It is important to mention that most often, the reagents utilized are generally expensive, toxic, and could lead to allergic effects. Meanwhile, the preparation of hydrogels using a chemical process with high energy beams (ex: gamma rays/electron beams) has gained significant attention due to the formation of pure hydrogel products. Thus, when compared to the gamma ray technique, the electron beam irradiation showed immense potential due to its rapid speed and a particular beam direction during the production of hydrogels. However, one of the complications in preparing hydrogels of natural polymers in an aqueous solution by irradiation is the higher possibility of chain breakage reactions than crosslinking reactions. Lately, phenolic compounds have been conjugated to natural polymers trailed by crosslinking with chemical reagents or irradiation. Against this background, using electron beam irradiation, porous and non-porous Pec-based hydrogel systems were fabricated using a combination of Pec and 5-hydroxytryptophan (5-HTP) with or without a surfactant for the delivery of tetracycline (TET). The drug loading for the porous hydrogel and non-porous hydrogel systems was 103 and 77 mg/g, respectively. The drug release from the non-porous hydrogel was comparatively less when compared with the porous hydrogel and showed higher amounts and a faster rate. Consequently, the biocompatibility and non-toxicity of both hydrogels were within the acceptable limits [52].

**Figure 3:**
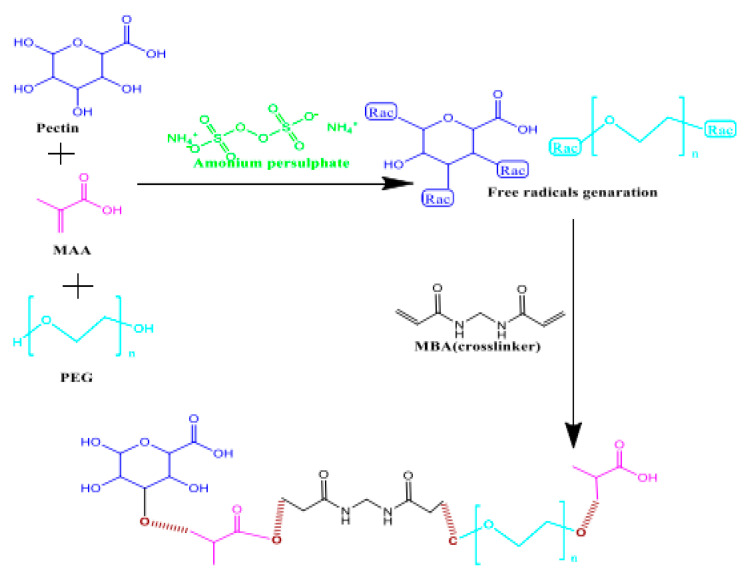
Scheme showing the formation of Pec-g-PEG-MAA hydrogels. Reproduced with permission [53].

Abbasi et al. reported an efficient hydrogel-based controlled drug delivery system where Pec was grafted with polyethylene glycol (PEG) and methacrylic acid (MAA) via free radical polymerization (Figure 3) for the treatment of ulcerative colitis. Sulfasalazine (SZ) was used as a model drug in order to load the designed hydrogel systems. The swelling and release studies revealed that the hydrogels could release drugs explicitly at colonic pH. The toxicological studies revealed that they were safe in mouse animal models [53].

**Figure 4:**
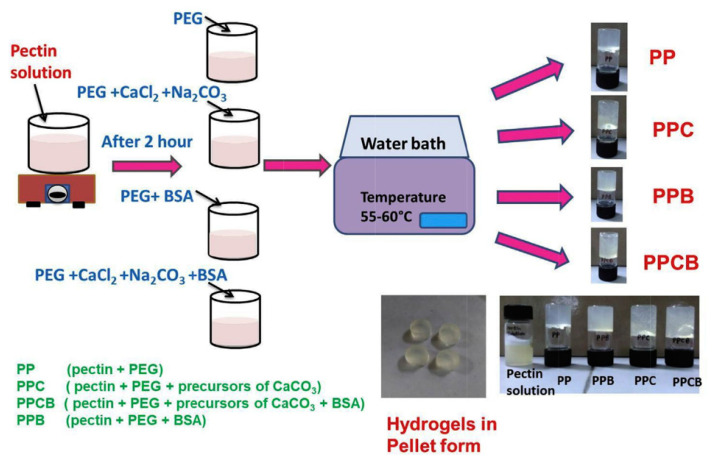
Schematic representation involved in the preparation of Pec-based hydrogels. Reproduced with permission [54].

Generally, protein administration via the oral route has continued as an appealing strategy due to less pain, superior suitability, and improved patient compliance. Herein, calcium carbonate microparticles (CaCO_3_) were mineralized in situ in a Pec/poly(ethylene glycol) (PEG) hydrogel blend to shield and release the bovine serum albumin (BSA) protein drug (Figure 4) at the specific colon site. The BSA encapsulation efficiency for the blended hydrogels was around 98%. In vitro swelling and protein drug release studies of a-based hydrogel revealed the drug carrier’s ability to release protein for around 9 h at the colon site [54].

In another work, Pec based lactic acid (LA) hydrogels were fabricated using free radical polymerizations. As the concentrations of Pec and lactic acid increased swelling, drug loading and drug release were observed, whereas methacrylic acid (MAA) showed the opposite effect. The loaded oxaliplatin (OL) to the developed hydrogel systems displayed around 49% of entrapment efficiency and substantiated with 18, 41, and 47% of drug release at pH 1.2, 6.8, and 7.4, respectively. Based on the MTT assay, the drug loaded hydrogels confirmed controlled inhibition against HCT-116 and MCF-7 cells [55].

**Figure 5:**
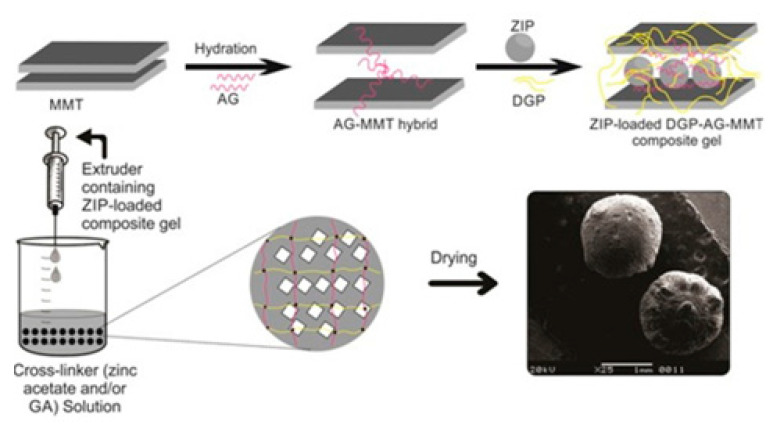
Scheme for the formation of Pec-based MMT hydrogels and ZIP loading. Reproduced with permission [56].

Other research studies involved amine grafted high methoxy Pec-arabic gum (AG) incorporated montmorillonite (MMT) composite hydrogels for ziprasidone HCl (ZIP) delivery (Figure 5). The hydrogels showed around 39–64% of entrapment efficiency for ZIP and proved that slow ZIP release up to 8 h indicated excellent gastroretention ability and biodegradable properties. Overall, the designed hydrogels, fabricated using green synthesis, have the potential to be used as an effective intragastric drug carrier for the treatment of schizophrenia [56].

Usually, thiolated polymers are used in drug delivery applications due to their better permeation features, which provide superior bioavailability. However, the thiolation process is time intense due to a series of chemical reactions. To address such drawbacks, 2-thiobarbituric acid (TBA) was integrated onto the Pec hydrogels via noncovalent interactions. Importantly, due to the incorporation of TBA, the Pec hydrogels showed an additional flexible nature and their fragmentation delayed from 4 h to 4 days. Furthermore, the loaded theophylline (THP) drug to the hydrogel systems showed a loading capacity of 30 mg/g and showed a controlled drug release of up to 400 min [57].

**Figure 6:**
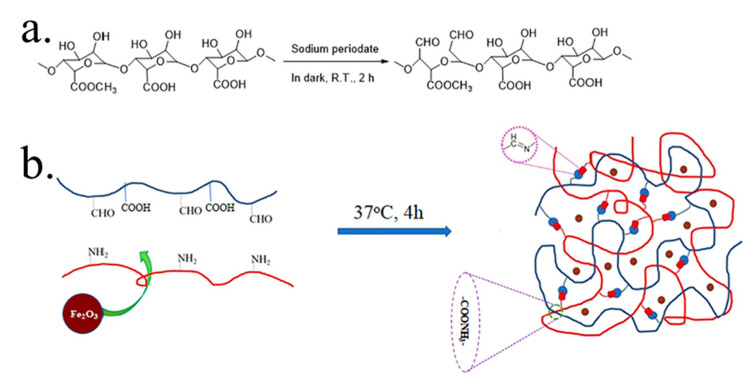
Proposed scheme (**a**) oxidation reaction and (**b**) hydrogel formation process. Reproduced with permission [58].

It is significant to mention that injectable hydrogels with self-healing properties are important for drug delivery. One such work was reported by Li et al. by varying the ratios of oxidized Pec/chitosan (CS) to nano iron oxides (n-IO) (Figure 6), which showed remarkable injectable, self-healing, biocompatible, and anticancer features for the loaded 5-flurouracil (5-FU) drug with a drug release profile of more than 12 h [58].

Other work have reported a combination of hydroxypropyl methylcellulose (HPMC) and Pec-based hydrogels fabricated by the free radical polymerization process. Furthermore, loaded galantamine hydrobromide (GHBr) showed varied drug entrapment efficiency in the range from 63 to 95% based on the swelling capacity of the designed hydrogel systems. The formulated hydrogels exhibited pH dependent behavior and intelligent response to environmental conditions by controlling the drug release for around 37 h. Additionally, the toxicity studies conducted on albino male rabbits were proven to have safe efficacy for hydrogel systems [59].

Gazzi et al. developed a Pec hydrogel comprising imiquimod loaded polymer nanocapsules for melanoma treatment. The loaded imiquimod (IQ) drug content was around 0.52 mg/mL and in vitro release study disclosed 63% of imiquimod release from the hydrogel systems for 2 h, while 60% of the drug was released after 8 h, followed by controlled release up to 24 h. In addition, the designed hydrogel systems displayed superior adhesiveness and a higher penetration of the drug inside the skin was observed [60].

Another work reported pH responsive hydrogels containing zein protein nanoparticles (ZPN) and Pec biopolymer for the encapsulation of doxorubicin (DOX) and release studies. It is important to note that the nanoparticles aided in the formation of complete gel networks for the loading of DOX. Interestingly, DOX loaded hydrogels showed better cytotoxicity effects against cervical cancer cell lines. In addition, the designed hydrogels were responsible for the pH dependent release of DOX to the cytosolic acid environment of HeLa cells. Altogether, this unique combination of zein and Pec-based hydrogels was favored with controlled release, improved shell life of the drug, and are capable of creating an intrinsic environment for the drug [61].

Keeping our focus on ulcerative colitis (UC), pH sensitive and enzymatically triggered hydrogels containing Pec and polyacrylamide (PA) were used to load budesonide (BUD). Based on the gel fraction and swelling behavior of the optimized hydrogel formulations revealed 80% of encapsulation efficiency and 8.8% of drug loading capacity. In vitro release of BUD from the hydrogel unveiled a sustained release behavior with non-fickian diffusion mechanism over a time period of 1400 min. However, future studies such as stability, cytotoxicity and in vivo studies should be of focus to prove the potent drug delivery systems [62].

In another work, cellulose nanofiber (CNF)-alginate (Alg)-Pec-based hydrogel systems was developed for breast cancer treatments. Furthermore, the 5-FU drug was loaded to the hydrogel system and for the different hydrogel formulations, the encapsulation efficiency varied from 62 to 76%. The developed hydrogel formulations were facilitated by the initial burst release, followed by controlled drug release up to 24 h. Remarkably, the developed hydrogel systems enabled modulating the viability of breast tumor cells and inflammasome activities [63].

Similarly, in another work using nanocellulose fibers (NFs), low methoxy Pec and sodium alginate-based biocomposite hydrogels were synthesized. For the designed biocomposite-based hydrogel, clindamycin hydrochloride (CH) was loaded. By varying the ratios of hydrogels and drug, it could be possible to attain 82–94% of drug loading content. In vitro drug release profile showed 30–38% of drug release for the first 3 h and 100% of drug release was observed for 48 h. In addition, a cell viability study revealed superior cytocompatibility for human keratinocyte cells. It is worthwhile mentioning that these kinds of formulations are highly required in transdermal drug delivery. However, additional studies need to be carried out for the complete evaluation of the potential pharmaceutical applications toward the designed hydrogel systems [64].

One of the significant properties of self-healing hydrogels is pH stimuli and aid to protect the drug from being destroyed until it reaches the target site. Thus, in this report, through the Diels–Alder reaction, Pec/chitosan (CS) hydrogel systems were fabricated. The 5-FU drug was loaded to the designed hydrogel systems, which proved to have superior loading efficiency and sustained drug release profiles. As expected for the series of the designed hydrogel formulations, varied drug loading efficiency was observed from 53–65%. In vitro drug release displayed 30% of drug release for the first 4 h, followed by sustained drug release up to 12 h. Significantly, the developed hydrogel systems were cytocompatible for fibroblast L929 cells [65].

**Figure 7:**
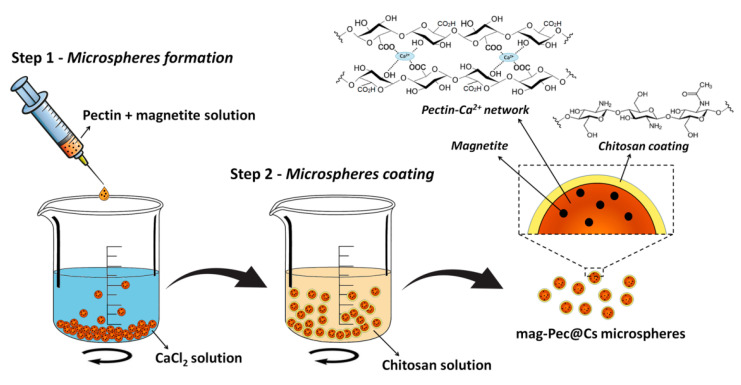
Scheme for the illustration and formation of magnetic Pec microspheres coated with chitosan. Reproduced with permission [66].

Lemos et al. developed magnetic (Mag) hydrogel microspheres using Pec coated chitosan for smart drug release. Herein, magnetic Pec microspheres attained by ionotropic gelation trailed by polyelectrolyte complexation with chitosan (Figure 7). In this study, metamizole (MTZ) was loaded and showed an encapsulation efficiency of 85%. In vitro drug release was performed at pH 1.2 and 6.8, which suggested a pH dependent drug release profile. Evidently, at pH 6.8, the drug release was favored by attaining 75%, even after 12 h. The utilization of the magnetic field amplified the drug release to 91% at pH 6.8, suggesting the role as magnetic reliant [66].

Another motivating work report involves new injectable and self-healing hydrogels fabricated using aldehyde terminated Pec with poly(N-isopropylacrylamide-stat-acylhydrazide) for an improved anticancer, DOX drug release property. In vitro and in vivo studies showed that the hydrogel had good biocompatibility, biodegradability, reduced drug toxicity in living bodies, and displayed controlled drug release behavior as synergetic anti-tumor drug delivery carriers [67].

It is well-known that amphiphilic (Amp) polymer systems have gained more interest due to their benefit in increasing drug penetration over the skin. Thus, focusing on this special aspect, an amphiphilic alkylated Pec through glycidyl tert-butyl ether functionalization was carried out to obtain hydrogels for fusidic acid (FSA) diffusion for topical treatment. The hydrogels were constructed via ionic interactions of negatively charged Pec and positively charged crosslinkers with varied 93–95% of FSA drug loading capacity. The swelling percentage of alkylated Pec hydrogels was lower than that of native Pec, resulting in a slower fusidic acid release up to 185 min. The effect of pH on the swelling rate and drug release was also studied, with outcomes showing that higher pH increased the swelling percentage and drug release. Interestingly, in vitro co-related with HaCaT cells displayed significantly less cytotoxicity, however, further extensive investigation is required [68].

Cai et al. reported low methoxyl citrus Pec (LMP) hydrogels as an actual drug carrier to load curcumin (CUR) for colon targeted delivery systems. The encapsulation efficiency (EE) for the designed hydrogels varied from 37 to 40% and loading capacity (LC) of 2.5–3.0%, respectively. Due to the better EE, the designed hydrogels displayed improved texture properties, inhibited premature release in the gastrointestinal (GI) tract, and were able to release the drug in the colon area at a faster rate, which attained a drug release rate at around 20 h [69].

In another work, Pec-Alg-based zinc (Zn) alginate hydrogel particles obtained based on callus culture Pec with varied structures were formed. The development of a Pec-alginate interpenetrating network was confirmed by the increase in hydrogen bonds between Pec and alginate. Usually, grape seed (GS) extract displays an anti-inflammatory outcome for inflammatory bowel disease (IBD) and thus grape seed extract was loaded to the designed hydrogel systems and revealed 95% of encapsulation efficiency. The drug release studies were carried out at different pH conditions and showed drug release specifically in colon conditions, which might be alternative candidates for colon targeted drug delivery systems [70].

Other findings reported functionalized kappa-carrageenan (K-CRG)-Pec hydrogel patches for the treatment of buccal fungal treatment. Herein, kappa-carrageenan-g-acrylic acid was surface functionalized with thiolated agents. Furthermore, in an ex vivo mucoadhesion study, a swelling test was extensively carried out to prove the applicability of the hydrogel patches. The triamcinolone acetonide (TA) was encapsulated within the poly (lactic-co-glycolic acid) nanoparticles. The EE and drug loading were 79% and 10%, respectively. The in vitro drug release parameter showed that the amount of drug release was around 3.28 mg/g polymer after 7 h. The cell culture studies on the hydrogel patches revealed that none of the patch formulations were toxic. All of these interesting findings suggest that novel thiolated grafted hydrogel patches could be utilized for buccal drug delivery systems [71].

**Figure 8:**
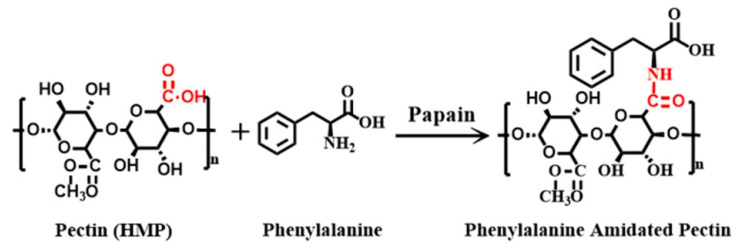
Scheme for the formation of phenylalanine amidated Pec. Reproduced with permission [72].

Another work showed that to enhance the gel properties, Pec was additionally modified with phenylalanine (Phe) using an ultra-low temperature supported enzymatic process (Figure 8). Thus, the designed hydrogels exhibited better mechanical properties and superior water holding capacity. Due to the good gel features, matrine (MT) was loaded and showed sustained release properties with swelling properties. In cases of poor drug release, the ultra-low temperature enzymatic process might be a viable approach [72].

Regulating the optimal drug concentration and controlling drug release from hydrogels necessitate a large number of experiments and is overall expensive. To address such issues, in this work molecular dynamics (MD) stimulations were used to envisage the actual drug concentration to load on the LMP-based hydrogels, which allow for structural integrity and controlled drug release. When compared to other samples, Pec hydrogels loaded with 30 mg procaine (PRO)/g had a low hydrogel degradation rate of 0.001 g/min and a controlled in vitro drug release, releasing all 30 mg of the loaded PRO from the 670 mg hydrogel in 24 h [73].

Another study examined the fabrication of the Pec/chitosan nanoparticle (PEC/CSNP) beads as nanocarriers by encapsulating quercetin (QR) to overcome the solubility and sensitivity issues. The fabricated hydrogel beads exhibited 34–56% of EE and 12–24% of loading capacities. The prepared beads were able to release quercetin in a sustained release pattern up to 480 mins, as demonstrated in an in vitro drug release study. Furthermore, an in vitro cytotoxicity study revealed that the designed beads displayed a cell viability above 80% on the L929 cell line [74].

**Figure 9:**
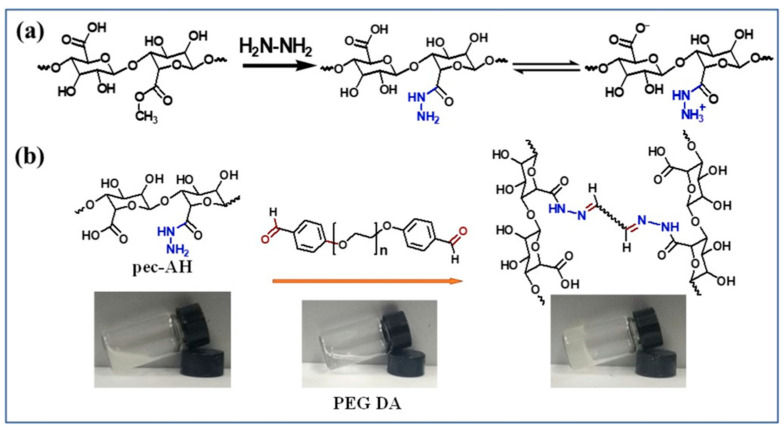
(**a**) Scheme for the synthesis of Pec-AH and (**b**) formation of the Pec-AH and PEG-DA hydrogel. Reproduced with permission [75].

In another part of the work, a self-healing hydrogel was created by cross-linking Pec acylhydrazide (Pec-AH) with polyethylene glycol dialdehyde (PEG-DA) (Figure 9), and its use as a doxorubicin (DOX) delivery carrier for operative tumor treatment was examined. Significantly, the hydrogels demonstrated excellent in vitro and in vivo biocompatibility and biodegradability, with controlled drug release for around 50 h at varied pH conditions due to their microporous structure. The xenograft CT-26 tumor model in the mice trial exposed that the DOX-loaded hydrogel could prevent tumor growth associated with the direct injection of DOX and eliminate the associated drawbacks [75].

Another study attempted to develop Pec-based layered zinc hydroxide (LZH) hydrogels comprised of baclofen. Through in vitro studies, they revealed that Pec-LZH containing baclofen (BFN) displayed a lower release rate when compared with BFN loaded with LZH. An addition, the MTT study suggests that for the HFFF2 cells, the prepared hydrogel system was biocompatible at 1.564–25µg/mL doses. The developed hydrogel beads appear to be promising as efficient carriers for targeted delivery to the colon [76].

**Figure 10:**
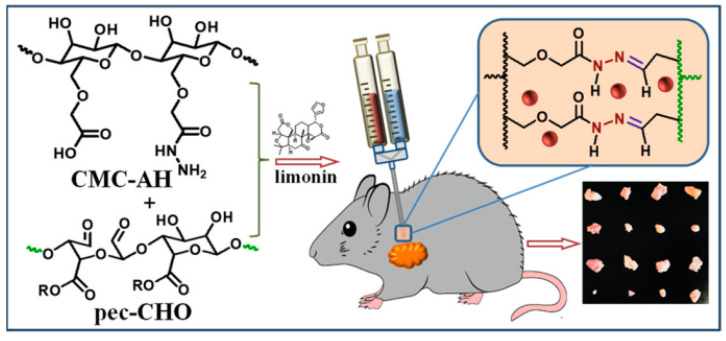
Scheme for the limonin loaded hydrogels for lung cancer treatment. Reproduced with permission [77].

Due to the lack of accurate drug targets, lung cancer is considered as the most common malignant tumor. This study involves limonin (LM), which prevents proliferation and encourages apoptosis in lung adenocarcinoma cells by directing a specific high expressed TMEM16A ion channel. Furthermore, a new class of self-healing hydrogels was created using acylhydrazide functionalized carboxymethyl cellulose (CMC-AH) and oxidized (Pec-CHO) (Figure 10) to decrease limonin’s adverse effects on the body. The hydrogels demonstrated rapid gelation, better biocompatibility, and long-term limonin release up to 12 h. The limonin-loaded hydrogel expressively inhibited the development of lung adenocarcinoma in xenograft mice [77].

Other work reported with photo-crosslinkable PC-CN formed by functionalizing the polysaccharide Pec (PC) with the photo-responsive cinnamic acid hydrazide (CNH). The photo-crosslinked hydrogel was then assessed as a carrier for the encapsulation of aspirin (AS). The developed hydrogels demonstrated better potential as a drug carrier, allowing for controlled drug release up to 60 h at different pH conditions by improving both the degree of cinnamic functionality and the photo-curing time [78]. Overall, Table 1 summarizes the various types of Pec-based hydrogel systems, the drugs used, and their key features involved in drug delivery applications.

## 7. Toxicity Concern of Pec-Based Hydrogels

Due to the excellent properties of Pec such as its biocompatibility, biodegradability, and non-toxic nature, researchers have been attracted to utilize Pec-based hydrogel systems in drug delivery fields. Prominently, for drug delivery applications, the developed hydrogel systems must be nontoxic while being able to perform their functions in response to the host’s action. Based on some research findings, toxic materials can show negative impacts on the immune system. Some of the preliminary extensive research investigations have reported that Pec-based hydrogel systems have no such toxicity effects [52,75]. However, additional pre-clinical and clinical trials remain unresolved and must be prioritized as future prospects.

## 8. Conclusions and Future Perspectives

The present review mirrors based on the advancement of Pec-based hydrogels for drug delivery applications. The significance of Pec-based hydrogels is augmented by their unique functional groups, biocompatibility, biodegradability, easy gelling capability, low-cost, and simple modifications, which allow these systems to be astonishing candidates for the design and advance of potent drug delivery systems. It is significant to mention that by using a combination of other polymers and nanomaterials, the overall structural properties of Pec-based hydrogel systems can be considerably enhanced. Furthermore, more precise chemical modifications of Pec as well as their combination with other polymers or integration with other nanobiomaterials will enhance the overall structural behavior of Pec-based hydrogel systems and aid in tuning the interaction with the drug molecules at the molecular and nanoscale levels. With these substantial key features and the growing task of research groups on Pec-based hydrogel formulations, it could be projected that Pec applications in the drug delivery domain will expand in the near future. To this end, efforts should be increased to advance Pec-based hydrogel systems into clinical use, with the goal of dealing with regulatory problems, which are currently regarded as the main impediment.

## Figures and Tables

**Table 1:** Summary of various types of Pec-based hydrogel systems, drugs used and their key features.

Serial no.	Hydrogel Systems	Drugs Used	Key Features	Reference
1	LDL-Pec-Alg	CUR	Release profile of drug demonstrated with little slower rate in the simulated GI conditions, signifying role as oral drug delivery systems	[49]
2	MNP-Pec	DS	Suggesting swelling controlled diffusion mode of drug release profiles	[50]
3	SF-OP-PLLA	Vanco	Significant sustained release up to 192 h and non-toxic against hAD-MSCs	[51]
4	Pec-5-HTP	TET	Improved biocompatibility and non-toxicity are in tolerable limits	[52]
5	Pec-PEG-MAA	SZ	Drug release explicitly at colonic pH and toxicological studies revealed safe efficacy	[53]
6	Pec-CaCO_3_-PEG	BSA	Encapsulation efficiency was around 98% and drug release profile was around 9 h at the colon site	[54]
7	Pec-LA-MAA	OL	Controlled inhibition against HCT-116 and MCF-7 cells	[55]
8	Pec-AG-MMT	ZIP	Slow release and effective carrier for the treatment of schizophrenia	[56]
9	Pec-TBA	THP	Additional flexible nature and controlled release of drug	[57]
10	Pec-CS-n-IO	5-FU	Remarkable injectable, self-healing and biocompatible	[58]
11	Pec-HPMC	GHBr	Intelligent response to environmental conditions and toxicity studies conducted proved with safe efficacy	[59]
12	Pec	IQ	Displayed superior adhesiveness and higher penetration of the drug inside the skin	[53]
13	Pec-ZPN	DOX	pH dependent release cytotoxicity effects against cervical cancer cell lines	[61]
14	Pec-PA	BUD	Sustained release behavior and sustained release of 1400 min	[62]
15	CNF-Alg-Pec	5-FU	Enabled in modulating breast tumor cells	[63]
16	NF-Pec-Alg	(CH)	Superior cytocompatibility	[64]
17	Pec-CS	5-FU	Cytocompatible for fibroblast L929 cells	[65]
18	Pec-Mag-CS	MTZ	Improved drug release and pH dependent	[66]
19	Pec-PNIPAAm	DOX	Good biocompatibility and biodegradability	[67]
20	Amp-Pec-FSA	FSA	Proven with less cytotoxicity	[68]
21	LMP	CUR	Inhibit premature release in GI and able to release drug in colon area	[69]
22	Pec-Alg-Zn	GS	95% of encapsulation efficiency and drug release, specifically in colon conditions	[70]
23	Prc-K-CRG	TA	Cell culture studies revealed that none of the patch formulations were toxic	[71]
24	Pec-Phe	MT	Sustained release with swelling properties	[72]
25	LMP	PRO	Improved structural integrity	[73]
26	Pec-CSNP	QR	Sustained release and cell viability above 80% on the L929 cell line	[74]
27	Pec-AH-DA	DOX	Xenograft CT-26 tumor model in mice trial aided in preventing tumor growth	[75]
28	Pec-LZH	BFN	Low release rate and biocompatible	[76]
30	Pec-CMC-CHO	LM	Inhibited the development of lung adenocarcinoma in xenograft mice	[77]
31	Pec-CNH	AS	Controlled drug release up to 60 h at different pH	[78]

## Data Availability

Not applicable.
